# Detection of Lowering in Sport Climbing Using Orientation- Based Sensor-Enhanced Quickdraws: A Preliminary Investigation

**DOI:** 10.3390/s24144576

**Published:** 2024-07-15

**Authors:** Sadaf Moaveninejad, Andrea Janes, Camillo Porcaro

**Affiliations:** 1 Department of Neuroscience and Padova Neuroscience Center, University of Padova, 35128 Padova, Italy; camillo.porcaro@unipd.it; 2 Faculty of Engineering, Free University of Bozen-Bolzano, 39100 Bolzano, Italy; 3 Institute of Cognitive Sciences and Technologies (ISTC), National Research Council (CNR), 00185 Rome, Italy; 4 Centre for Human Brain Health, School of Psychology, University of Birmingham, Birmingham B15 2TT, UK

**Keywords:** machine learning, artificial intelligence, internet of things

## Abstract

Climbing gyms aim to continuously improve their offerings and make the best use of their infrastructure to provide a unique experience for their clients, the climbers. One approach to achieve this goal is to track and analyze climbing sessions from the beginning of the ascent until the climber’s descent. Detecting the climber’s descent is crucial because it indicates when the ascent has ended. This paper discusses an approach that preserves climber privacy (e.g., not using cameras) while considering the convenience of climbers and the costs to the gyms. To this aim, a hardware prototype has been developed to collect data using accelerometer sensors attached to a piece of climbing equipment mounted on the wall, called a quickdraw, which connects the climbing rope to the bolt anchors. The sensors are configured to be energy-efficient, making them practical in terms of expenses and time required for replacement when used in large quantities in a climbing gym. This paper describes the hardware specifications, studies data measured by the sensors in ultra-low power mode, detects sensors’ orientation patterns during descent on different routes, and develops a supervised approach to identify lowering. Additionally, the study emphasizes the benefits of multidisciplinary feature engineering, combining domain-specific knowledge with machine learning to enhance performance and simplify implementation.

## 1. Introduction

**Figure 1:**
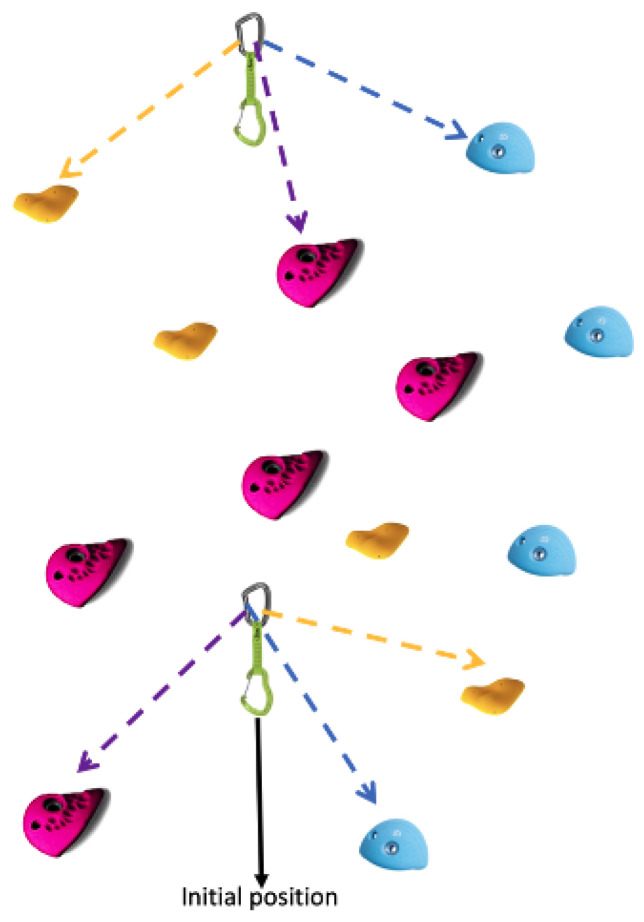
Different routes of the same line and corresponding quickdraws: climbers climb along the holds of the same color (called *route*) but attach themselves on hooks that are used for multiple routes, vertically along the wall (called a *line*).

Sport climbing has recently gained increased popularity internationally, both as a recreational activity and as a competitive sport. This sport can be performed either outdoors on natural cliffs or indoors in climbing gyms. In the case of indoor climbing, gyms provide walls that consist of several lines, each comprising multiple routes with varying difficulties, as shown in Figure 1. Additionally, climbing gyms must provide climbers with equipment and services that allow them to challenge themselves and improve their skills without compromising their safety. To achieve this, and similar to other sports (e.g., cycling or running), engineering and science have begun to assist in the development of sport climbing [1].

Climbing is known to offer extensive physical benefits, as it engages multiple muscle groups, including the upper body, lower body, and core, providing a comprehensive workout that enhances strength, endurance, and flexibility [2]. Furthermore, the cardiovascular health benefits of climbing are significant. The aerobic nature of climbing activities, especially during prolonged routes, elevates heart rates, contributing to improved heart health and increased stamina [2]. Additionally, climbing positively affects mental health by requiring focus, problem-solving skills, and the ability to overcome fear and anxiety. This mental engagement can help reduce stress, improve cognitive function, and foster a sense of accomplishment [2].

Resistance training, a fundamental aspect of climbing, enhances maximal strength, muscle hypertrophy, muscular power, and local muscular endurance, which are critical for climbing performance [3,4]. However, climbing also comes with potential risks and disadvantages [5]. Injury risks, such as falls or overuse injuries, are prevalent in both outdoor and indoor climbing environments. Common injuries include sprains, strains, and fractures, necessitating proper training [6] and safety measures to mitigate these risks [2]. The repetitive nature of the sport can lead to chronic injuries, particularly in the fingers, elbows, and shoulders, causing conditions like tendinitis and bursitis [2].

Additionally, while climbing can reduce stress, it can also introduce psychological strain, especially in competitive settings or when attempting difficult routes. The pressure to perform and the fear of falling can cause significant mental stress [2]. Moreover, outdoor climbing can have negative environmental impacts, such as damage to natural rock formations, vegetation, and wildlife habitats. Climbers must practice responsible climbing to mitigate these effects and preserve natural climbing areas [2].

The contribution of science to sport activities can take the form of collecting data from athletes using sensors embedded in electronic equipment such as smart watches, smart bands, fitness trackers, and smart phones [2,7,8]. These devices are attached to the body of the athletes (as in [8,9,10,11,12]). The corresponding measurements are time-series data used to visualize statistics of athletes and analyze their performance. Such analyses can be conducted either by a coach or through specific applications. Another method for acquiring data to analyze sport activities is based on cameras. In this approach, computer vision algorithms are developed to extract human two-dimensional (2D) pose sequences from video frames, relying on skeleton estimation of each person [13,14,15,16]. Instrumented climbing walls represent another approach for monitoring climbers, as inspected in [17,18]. Additionally, ref. [19] presents a sensor-equipped climbing wall with capacitive sensors embedded into climbing holds to detect touch by hands or feet, and [20] utilized force sensors to measure the load applied to holds during children’s climbing. Our approach, using accelerometer-based smart quickdraws, aligns with these studies by providing a practical and efficient solution for tracking climbing activities in gyms.

Accelerometers have been demonstrated to be effective at identifying a wide range of human activities [21]. They are often included in various systems reported in the literature for the identification of physical activity [22], energy estimation [7], and fall identification [23]. Accelerometers are well suited to extreme environments due to their relatively small size, battery-powered operation, and wireless capabilities [24]. The choice of accelerometers in climbing gyms is based on their energy efficiency and ease of integration into the quickdraws, making them economically feasible for large-scale deployment in climbing gyms. Accelerometers can provide valuable data for various climbing activities such as ascending, resting, falling, and lowering. This multi-purpose functionality is advantageous for climbing gyms aiming to enhance their infrastructure with a single type of sensor. While force sensors could offer additional insights, they come with higher costs and complexity for widespread implementation [24]. Additionally, the suitability of sensor types varies with different climbing styles. In lead climbing, where the climber attaches the rope to the current hook as they proceed, the “top” quickdraw is always the last one attached, necessitating force sensors for all quickdraws, which is impractical. In contrast, in top-rope climbing, the rope is already attached to the top quickdraw, which would indeed simplify the installation of force sensors. However, since top-rope climbing is primarily practiced by beginners, it would exclude a large part of customers in climbing gyms since many experienced climbers practice climbing in indoor gyms during the winter to prepare for the summer. Therefore, we concluded that accelerometers are a more practical choice for tracking various activities across both climbing styles, making them the preferred option for a large-scale deployment.

The data acquisition methods based on wearable sensors and cameras are not always desirable for climbers. Wearable sensors limit user convenience by requiring the climber to wear an extra device, and cameras can infringe on privacy or be perceived as a form of monitoring, which many climbers do not want to experience while practicing their hobby. Keeping these issues in mind, and similar to [25,26], this paper collects data from accelerometer sensors attached to a piece of climbing equipment mounted on the wall, called a quickdraw. Our study specifically focuses on the environment of a climbing gym, based on the requirements provided by the climbing gyms themselves. The primary objective is to understand the usage patterns of their clients without compromising their privacy or the authenticity of their climbing experience. To the best of the authors’ knowledge, sensor-enhanced quickdraws, hereinafter referred to as smart-quickdraws (s-qd), were first studied in [25]. Later, in [26], we utilized sensors in ultra-low power mode attached to the quickdraws to detect patterns in data during climbing. Using sensors in ultra-low power mode is necessary because, apart from climbers, another concern for data collection relates to the climbing gyms. There are a large number of quickdraws in a climbing gym, and regularly changing the batteries of the s-qds is expensive for the gym in terms of both time and costs. Hence, sensors attached to the quickdraws must be energy-efficient so that their batteries do not need to be replaced in the short term.

The data collected from climbers help both climbers and gyms in different ways. One goal is to improve climbers’ performance. To achieve this, different activities during climbing, such as ascending, resting, falling, lowering, and rope-pulling at the end of the climb, must first be detected. Then, the detected activities could be analyzed by experts in offline modes to provide recommendations for better performance or in online mode to improve safety by early prediction of risks. Another goal is to improve the infrastructure of the gyms based on climbers’ needs.

Machine learning has significantly advanced activity recognition and performance assessment in sport climbing. Various algorithms, including decision trees, logistic regression [10], k-means [26], Convolutional Neural Networks (CNNs) [25,27], random forests [28], and Artificial Neural Networks (ANNs) [29], have been employed to predict activities and calculate performance indicators [2]. Feature extraction and hierarchical- and threshold-based classification methods have further improved accuracy [2]. Approaches using logistic regression, random forest, and k-nearest neighbors are straightforward and effective for classifying multiple activities. Although powerful, ANNs can be slow to train and complex to implement. The real-time execution of ML algorithms can be hindered by the computational demands of high-dimensional feature processing [2].

In our approach, we determined the precise feature by integrating mechanical and robotics-related knowledge along with our observations in the climbing gym. This allowed us to skip the feature selection step, making our method more efficient than other machine learning methods. Moreover, with the precise feature, we use a simple decision tree model, making our method better than deep learning- and neural network-based approaches. By introducing our method, we not only present a good feature for detecting the lowering phase but also emphasize the importance of feature engineering using multidisciplinary science in machine learning and deep learning. This reduces model complexity and execution time, making it suitable for real-time applications in climbing gyms. Real-time analysis is crucial for detecting high-risk situations, enhancing climber safety and performance efficiency.

In this work, we investigated data from the lowest s-qds on the wall to detect the lowering phase. Lowering refers to the period from the time the climber finishes clipping the carabiner and starts to descend until they reach the ground and release the rope, allowing the belayer to start pulling and collecting the rope [25]. This reveals the end of each climbing activity and helps to separate sequential climbs for individual analysis. The authors in [25] introduced a hybrid system based on sensors and cameras to detect rope-pulling activity. Our proposed method is a supervised algorithm based on the classification of samples measured from a single sensor at the lowest position of the line.

However, using sensors in an energy-efficient mode and attaching them to the wall, instead of the climber’s body, introduces some challenges. Saving energy reduces the number of sensor samples and presents challenges in training machine learning models. Moreover, unlike algorithms based on wearable devices, which mainly collect data through a single sensor on the climber’s body, we need to deal with sensors mounted on the wall. In the following sections, all these points are addressed in detail.

### Contributions

The contributions of this paper are as follows:

1. We present the details of a data acquisition system based on accelerometer-enhanced quickdraws;
2. We develop an algorithm to detect the end of a climbing episode, called *lowering* and discuss its effectiveness;
3. We discuss the considered constraints when collecting data about climbing activities using sensors.

The remainder of this paper is structured as follows: we describe the details of the employed data acquisition system in Section 2 and the data collection phase in Section 3. Afterwards, in Section 4, the relevant features for lowering detection are extracted from sensor measurements. In Section 5, the selected features are used for detecting lowering, and the corresponding results are discussed.

## 2. Data Acquisition System

**Figure 2:**
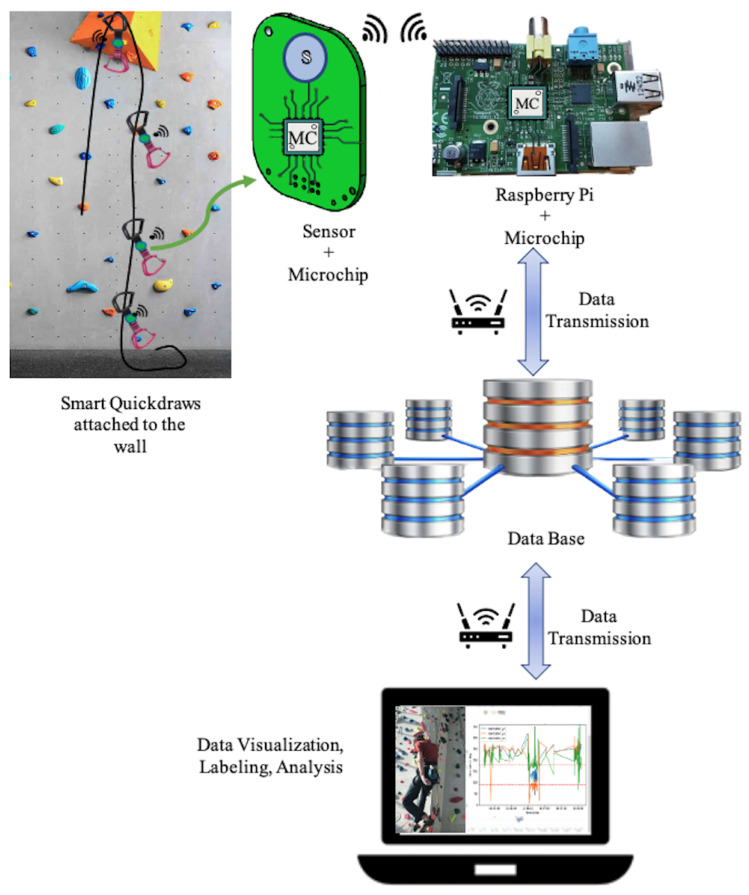
Data acquisition system.

Our data acquisition system in shown in Figure 2 and consists of two main parts: (a) smart-quickdraws to measure three-axis accelerations, and (b) a base station to receive data from sensors and forwards them to the database in a remote server similar to the data acquisition system in [26,30].

### 2.1. Smart-Quickdraw

The device referred to as the smart-quickdraw is a custom-designed circuit board affixed to the central part of the quickdraw’s strip. This circuit board is equipped with an accelerometer sensor that records the movements of the quickdraw and a microchip that manages the accelerometer and facilitates communication with the base station. Energy efficiency is crucial for our setup since replacing batteries for all smart-quickdraws in a climbing gym is both costly and labor-intensive. For this purpose, we chose the LIS3DH accelerometer from STMicroelectronics, known for its ability to function in ultra-low-power modes with smart wake-up and sleep functionalities. The microchip employed was the ATSAMR21G18A from Atmel, which integrates a microcontroller unit (MCU) and an RF transceiver. The accelerometer was programmed and controlled through firmware housed in the MCU, allowing the smart-quickdraw to communicate with the base station via its transceiver. Below are the primary configurations applied to the accelerometer sensors:

- Full scale: The LIS3DH can measure forces across a dynamically selectable range from ±2 g to ±16 g. Typically, in accelerometers, the smaller the range, the more sensitive the readings will be. Hence, we opted for a full scale of 2 g to enhance the sensitivity of the readings.
- Data rate: The LIS3DH is capable of recording accelerations at data rates ranging from 1 Hz to 5 kHz; we set our system at 10 Hz in inactive modes and 50 Hz in active modes to conserve energy.
- Output bits: The analog-to-digital converter (ADC) of LIS3DH supports 8, 10, or 12 bits of output data for operating in low-power, normal, and high-resolution modes. We selected an 8-bit output to maximize energy efficiency.
- Range and resolution: Accelerometers typically provide raw outputs that do not directly correspond to units like meters per second squared, necessitating an adjustment based on the chosen full-scale setting. For this particular sensor with an 8-bit signed output, the values range from −127 to 127. When adjusted for a full-scale range of ±2 g; a reading of 127 equates to +2 g of force, and −127 equates to −2 g. The resolution resulting from this scaling is approximately $\frac{2\;g}{127}\approx 16\;\mathrm{mg}$. Understanding this scaling and range is crucial for interpreting the output data from the sensor accurately.
- Noise reduction: Considering the considerable noise present at a 50 Hz sampling rate, we implemented a software averaging method that computes the average of every eight samples for each axis, which is then transmitted to the base station.
- Filter insignificant changes: The accelerometer continuously outputs readings at the designated sampling rate, irrespective of any movement. To conserve bandwidth, especially critical in low-power devices, the system is designed to omit transmitting sensor values that do not demonstrate significant changes on any axis. Specifically, a threshold of 15 units of the sensor’s output was set; changes less than this threshold are not sent to the base station. With a resolution of approximately 16 mg per unit, the 15 unit threshold is equivalent to the acceleration of 15×16mg=240mg, ensuring that only meaningful data are communicated.
- Grouping samples: In an effort to further reduce power consumption, the system was configured to send sensor readings in batches rather than individually. This grouping ensures that the radio of the smart-quickdraw remains off when no data are transmitted, thereby conserving energy. Typically, two samples are batched together unless the sensor is about to enter a sleep mode due to inactivity, at which point any remaining data are sent. While this batching may introduce a slight delay in data transmission to the base station, each sensor value is timestamped, which preserves the temporal accuracy essential for subsequent analysis.

### 2.2. Base Station

The base station includes a board with a microchip similar to that in the smart-quickdraw, mounted onto a Raspberry Pi. The Raspberry Pi acts as a robust CPU and hosts the application programming interface (API) that facilitates communication with the devices, along with a web-server and other potential services. Additionally, it communicates with its board via a serial line. The firmware on the board within the base station handles the translation of network signals used between the boards inside the base station and the smart-quickdraws.

### 2.3. Ultra-Low-Power Data Acquisition:

To maximize energy efficiency, the sensor within the smart-quickdraw is programmed by its CPU (within the MCU) to alternate between sleep and active operational modes. It is important to note that the CPU in the base station does not modify or directly manage the sensor settings. The following details how this is implemented:

- Sleep mode operations: When in sleep mode, the sensor remains static and collects data at a reduced rate of 10 Hz, conserving energy.
- Threshold-based activation: The output from the sensor is continuously monitored against a pre-set threshold defined in the smart-quickdraw’s firmware:
  1. If the measurements on all three axes are below this threshold (15 units of the sensor’s output), then these readings are deemed insignificant and are not processed by the CPU.
  2. Conversely, if data from any axis surpass the threshold, the CPU adjusts the sensor to a higher data acquisition rate of 50 Hz, commencing active data collection.
- Active mode processing: While active, the sensor compiles an average from every eight data points collected, which is then transmitted to the base station. Continuous monitoring ensures that if the averaged readings fall below the threshold for more than 0.8 s, the sensor is deemed inactive. If this state persists for 20 s, the CPU switches the sensor back to sleep mode, halting further data collection.

This configuration ensures that the sensor system only expends energy during periods of significant activity, thereby extending the life of its power source and maintaining efficient data capture when necessary.

## 3. Data Acquisition

One 27-year-old male climber participated in the experiment. He had been climbing for twenty years and had an advanced skill level, self-estimated as a 6b on-sight on the French rating scale of difficulty (FRSD). For the purpose of data collection, the participant was asked to climb three routes in the leading style on two different days. The three pre-selected routes had difficulty levels of 5c+, 6a+, and 6b+ (in the French grading system) and were chosen according to the climber’s skill level. The participant climbed at their usual speed, clipping the rope into every quickdraw, and was free to take resting time between climbs. The climber pulled the rope after lowering, and another person was responsible for belaying.

On the first day, he climbed each route five times. The order of climbing was (5c+, 6a+, 6b+), and this process was repeated five times. After three weeks, we asked the climber to repeat a similar experience, but the order of climbing was: (5c+, 5c+, 5c+), (6b+, 6b+, 6b+), and (6a+, 6a+, 6a+). The aim of changing the order of routes was to add different parameters, i.e., tiredness and provisioning, to the data collected from the same routes.

**Figure 3:**
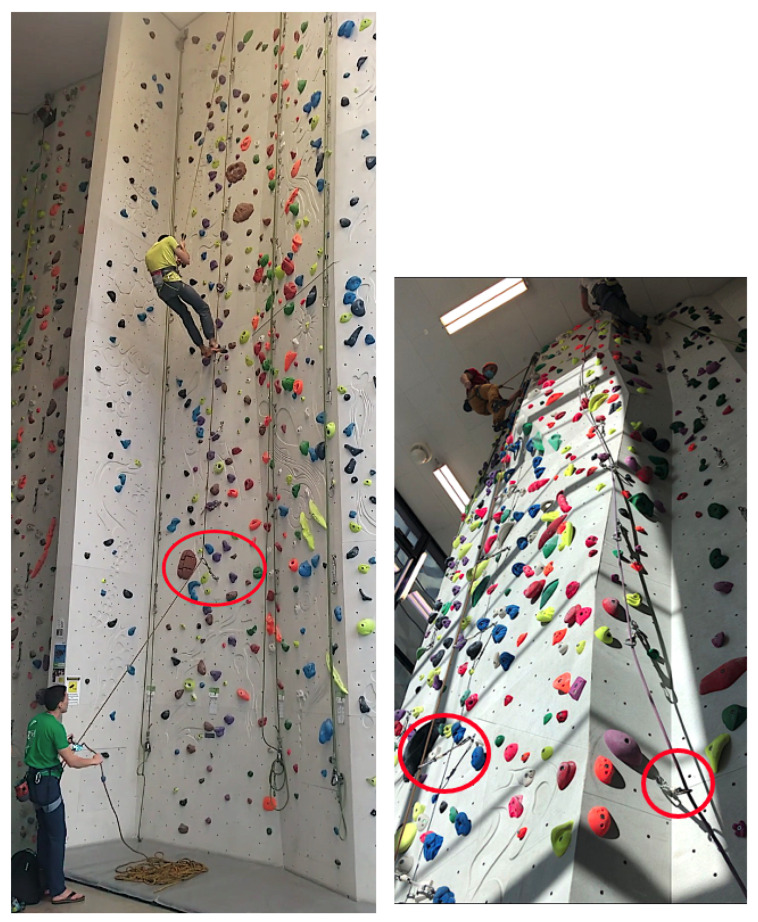
Orientation of the quickdraws and status of the climber with respect to the wall during lowering (**left**) and climbing (**right**). The first quickdraws are identifiable in this figure by the red circles around them.

As shown in the right panel of Figure 3, during climbing, the position of the quickdraw is highly dependent on several factors: the position of the belayer, the specific routes (position of the holds on the wall), and the skill or performance of the climber. These factors can cause significant variations in the orientation of the quickdraw. In contrast, during the lowering phase, the dynamics are considerably different. As shown in the left panel of Figure 3, the rope passing through the quickdraws is more constrained and primarily influenced by the gravitational force acting on the mass of the climber. The climber does not interact with the holds on the wall during lowering, which eliminates the impact of route difficulty on this phase. The only action performed by the climber is to hang and be lowered, which is a passive activity not affected by the climber’s skill level. Thus, the lowering phase can be considered an action of a heavy mass being lowered consistently by gravity, with minimal variations caused by external factors. In this context, the weight of the climber is only relevant because the weight of the belayer must not be too much lower than the weight of the climber. If the belayer’s weight is too low, they could be lifted into the air during a fall and might lose control of the rope [27,31,32].

All eight quickdraws in the line were enhanced with sensors. The climber started climbing by attaching the rope to the first quickdraw. The ascent ended after clipping the rope to quickdraw number eight, and lowering then started.

Employing sensors in ultra-low-power mode enhances energy saving but significantly reduces the number of samples. Considering this, we need to carefully understand the sensor functionality to analyze the received samples and identify the most informative ones for our objective.

## 4. Feature Engineering

The sensor embedded in smart-quickdraw works in ultra-low-power mode; hence, the s-qd does not continuously send data to the base station. Instead, it transmits samples when it is in active mode and the change in the movement of the corresponding quickdraw exceeds a certain threshold. To detect lowering, in our experiment, the first two sensors of the line were sensor-enhanced and *i* refers to the position of the s-qds in the line. During climb *c*, the sensor at position *i* transmits samples with timestamp $t_{i_{k}}$:

(1) $${T_{i}}^{c}=\left\{t_{i_{k}}|k⩾1\right\}$$

where *k* refers to the index of the samples and ${T_{i}}^{c}$ is the set of timestamps of all samples transmitted from s-qd at position *i* to the base station. These samples are measured from the time when the s-qd is clipped untill the climber’s movement still causes tangible changes in the acceleration of the s-qd.

After visually keeping track of about 100 climbers in the gym, we assumed that the most relevant information for detecting lowering was the orientation of the first s-qd that the climber clips the rope to. This quickdraw could be either in the first or second position in the line. Accordingly, we first calculated the orientation of the first clipped s-qd in three planes and then extract statistical features from the orientation in each of the three planes, i.e., mean, max, standard deviation, etc.

### 4.1. Orientation of s-qd

**Figure 4:**
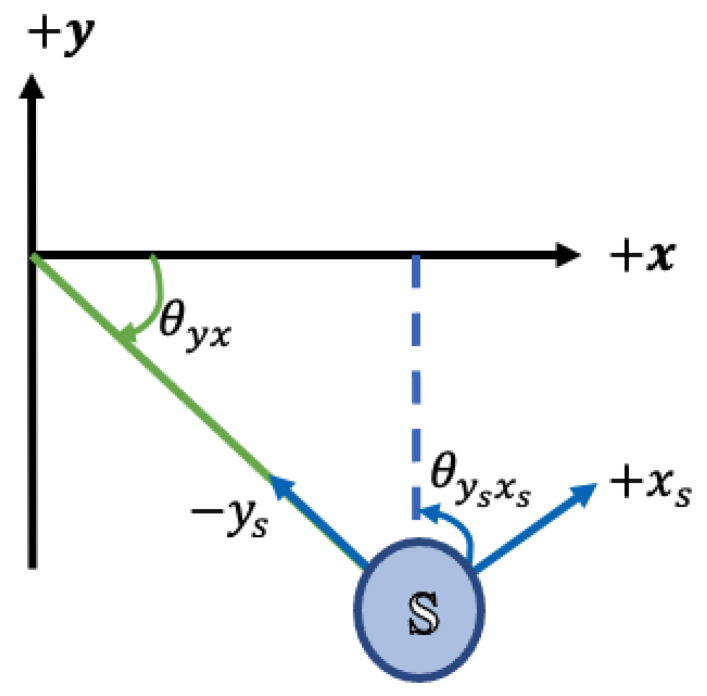
x, y, and z directions of three-axis accelerometer sensor with respect to the climbing wall.

The feature that can provide us with meaningful information about the position of the s-qd and helps us to distinguish the lowering activity is the orientation of the quickdraws during climbing. To use such a feature, we need to calculate the orientation of the quickdraw from three axes to axis acceleration measured by its sensor. To this aim, we evaluated the discrete orientation of the s-qd for each sample independent of the prior and posterior ones. Moreover, samples were analyzed in three two-dimensional (2D) coordinate plans to detect whether the s-qd was upward or downward, right or left, and forward or backward. Hence, for each sample, we have three values $\left(\theta_{y_{s}x_{s}},\theta_{y_{s}z_{s}},\theta_{x_{s}z_{s}}\right)$ corresponding to the orientation of the sensor in $y_{s}x_{s}$-plan, $y_{s}z_{s}$-plan, and $x_{s}z_{s}$-plan, respectively. Here, $\left(x_{s},y_{s},z_{s}\right)$ refers to the three-axis acceleration of the sensor in its own coordinate system. Orientations of s-qd with respect to the coordinate system of the sensor must be mapped to the coordinate of the wall (x,y,z), as shown in Figure 4; thus,

(2) $$\theta_{yx}=\theta_{y_{s}x_{s}},\;arctan\left(\frac{-y}{x}\right)=arctan\left(\frac{-y_{s}}{x_{s}}\right)$$

(3) $$\theta_{yz}=180^{\circ }-\theta_{y_{s}z_{s}},\;arctan\left(\frac{y}{z}\right)=arctan\left(\frac{y_{s}}{-z_{s}}\right)$$

(4) $$\theta_{xz}=180^{\circ }-\theta_{x_{s}z_{s}},\;arctan\left(\frac{x}{z}\right)=arctan\left(\frac{x_{s}}{-z_{s}}\right)$$

**Figure 5:**
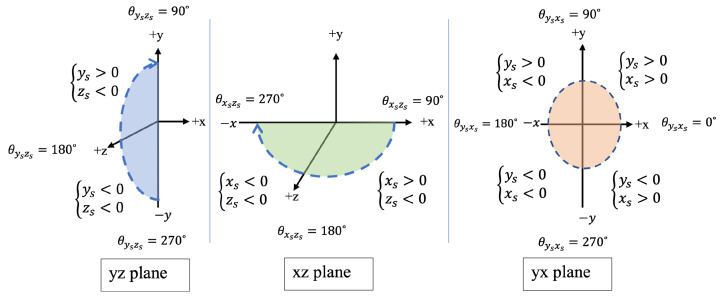
Mapping orientation of a smart-quickdraw to the wall.

The outcome of such a conversion between coordinate systems is shown in Figure 5. For each s-qd, $\theta_{y_{s}x_{s}}$ and $\theta_{x_{s}z_{s}}$ are sufficient to determine whether the s-qd is oriented upward/downward and left/right, respectively. $\theta_{y_{s}x_{s}}$ corresponds to the vertical orientation, which is caused by the difference in vertical force applied to the s-qd by the climber and the belayer. $\theta_{x_{s}z_{s}}$ indicates the horizontal orientation, showing whether the s-qd is positioned to the right, left, or middle, based on the force applied to the quickdraw by the belayer.

### 4.2. Orientation of s-qd during Lowering

**Figure 6:**
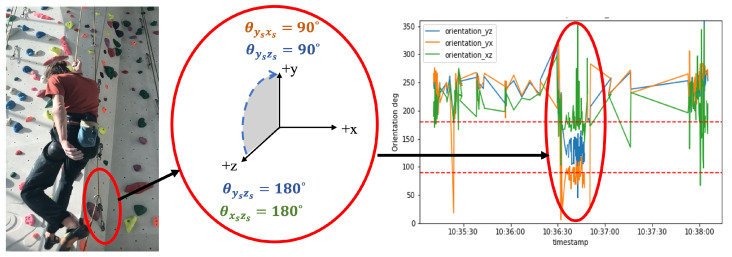
Orientation of the first smart-quickdraw during lowering. The first quickdraw is identifiable in this figure by the red circle around it.

We can use orientation to detect lowering and, consequently, the end of a climb. Lowering is an activity which lasts several seconds; on one side, there is belayer on the ground holding the rope; on the other side, the climber is hanging on the rope while being lowered. Hence, tension applied to the rope from the climber is more than that applied by the belayer. Consequently, as long as the climber is lowering, the first quickdraw has an upward orientation orthogonal to the wall, as seen in Figure 6. In other words, the orientation of the first s-qd must be between ($90^{\circ }$ and $180^{\circ }$) in the $y_{s}z_{s}$-plan, around $90^{\circ }$ in $y_{s}x_{s}$-plan, and around $180^{\circ }$ in $x_{s}z_{s}$-plan. keeping this in mind, the lowering is evident from sensor measurements in Figure 6. It worth mention that, due to the presence of the wall, the orientation of the s-qd in the $x_{s}z_{s}$-plan is limited between ($90^{\circ }$ and $270^{\circ }$) and the values out of this range are a sign of change in coordinates of the sensor, which is caused by twisting.

Based on these assumptions, a vector of orientation could be calculated from samples measured by the lowest s-qd in a period between the moment when the s-qd was activated through clipping untill it was still and stopped sampling.

### 4.3. Resampling

**Figure 7:**
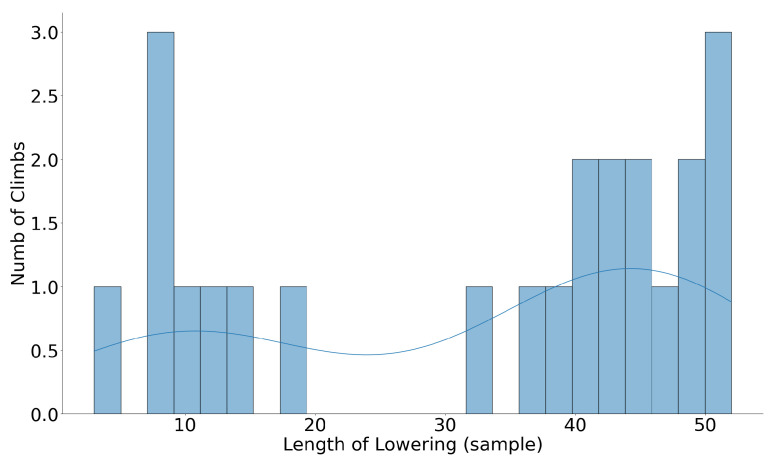
Duration of lowering in different climbs before resampling.

**Figure 8:**
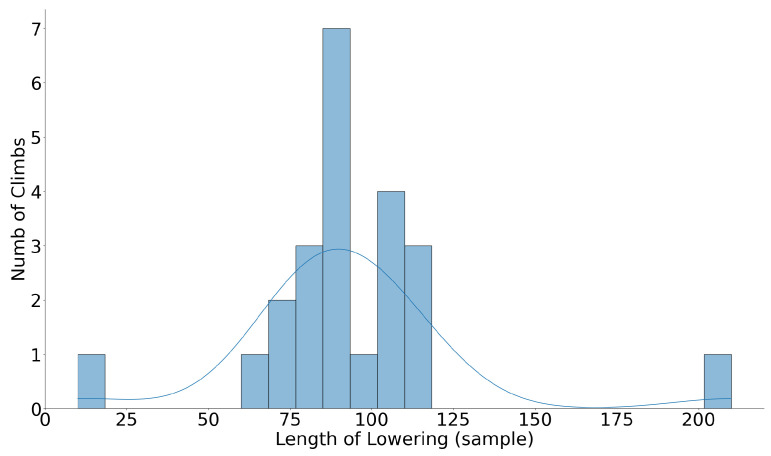
Duration of lowering in different climbs after resampling.

Different climbs have different duration and a different number of samples. Therefore, before classification, we used resampling to have the same number of samples for all climbs. Figure 7 and Figure 8 show the number of samples during lowering before and after resampling the climbs to one minute, respectively. Before classification, features were optimized in two steps: 1—feature scaling, and 2—feature selection.

## 5. Classification

The lowering activity is a temporal event that may be detectable from data measured through sensors. In this regard, sliding windows were used to transform each interval of time-series data into an appropriate feature vector (orientation along three axes and corresponding statistics). As a result, samples from s-qd during each climb were converted to several sliding windows with or without overlap. For this classification task, we utilized stratified k-fold cross-validation [33], ensuring that each fold contained roughly equivalent proportions of class labels, thus maintaining the integrity and balance of our dataset. Specifically, this study employed stratified ten-fold cross-validation with three repetitions over sliding windows with different lengths to find the optimum length for windows, which corresponds to the best performance of the classifier across different data splits.

The decision tree classifier was selected for its efficacy in handling the nonlinear relationships typical of sensor data from physical activities. We assigned the label for a window to be *lowering* only if at least 90% of its samples within that window were identified as such; otherwise, the window was labeled as *not_lowering*. We also implemented a two-sample overlap between subsequent windows to maintain temporal continuity.

**Figure 9:**
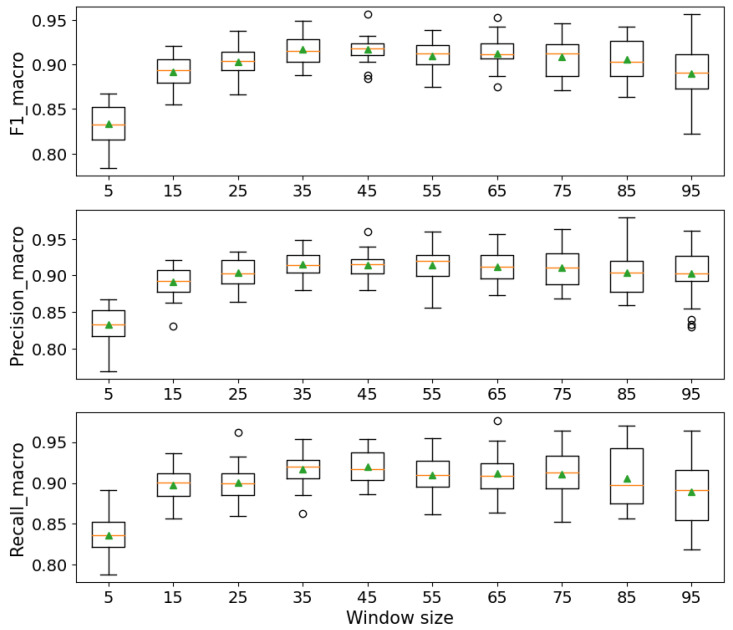
Classification of lowering and not-lowering for different window sizes.

Figure 9 illustrates the effectiveness of various window sizes on the classification results. The optimum window size was identified to be 45 samples, at which precision, recall, and F1 scores all exceeded 90%. The best classification outcomes were consistently achieved at this window length, beyond which no significant improvement in classifier performance was observed. As shown in Figure 8, after resampling, the length of lowering in almost all climbs exceeds 60 samples. This finding supports the selection of a 45-sample window as approximately half the average number of samples representing the lowering phase after resampling, indicating its adequacy in capturing the significant activity details without redundant data, thus enhancing the classifier’s accuracy and efficiency.

## 6. Discussion

This section discusses the achieved results of this paper along the following criteria: sensor accuracy and precision, calibration issues, and participant variability.

### 6.1. Sensor Accuracy and Precision

The sensors’ ability to accurately detect orientations was extensively validated before their installation in the climbing gym. This validation involved the precise alignment of sensors in predefined orientations, with outputs verified against calculated theoretical values. Such rigorous pre-installation testing assured high reliability in capturing accurate orientation data essential for the subsequent analysis of climbers’ movements.

Further evaluation of sensor accuracy and precision in detecting the lowering phase was conducted through the analysis of classification outcomes. Figure 9 demonstrates the performance metrics—precision, recall, and F1 scores across various window sizes. Optimal performance is observed at a window size of 45 samples, where all scores exceed 90%. These results indicate a high level of accuracy in the sensors’ ability to correctly identify lowering events, with minimal misclassification. The insights gained from Figure 9 provide a benchmark for researchers and developers aiming to implement similar sensor-based detection systems, illustrating how different window sizes affect classification outcomes. This analysis aids in optimizing the balance between responsiveness and accuracy, thereby enhancing the system’s reliability and applicability in real-world scenarios.

Despite the high scores indicating robust sensor performance, it is crucial to acknowledge potential sources of error that could still influence precision and accuracy, such as sensor noise and the reduced sampling rate due to the sensors’ ultra-low power settings. These factors may lead to false positives, where non-lowering activities are misclassified as lowering, or false negatives, where actual lowering activities go undetected. Understanding these limitations, is vital for system developers to design more effective data acquisition and processing strategies. Such strategies should aim to minimize error rates while maintaining energy efficiency, providing a practical blueprint for deploying these systems in various sporting or monitoring applications. Previous studies, such as Andrić et al. (2022), highlight similar challenges in sensor-based activity recognition, emphasizing the importance of balancing energy efficiency with accuracy to minimize error rates in practical applications [2].

### 6.2. Calibration Issues

To avoid the need for calibration, we want to mention that during installation, all sensors were installed with the same physical orientation, i.e., with their upper part towards the ceiling of the climbing gym. Moreover, as depicted in Figure 2, the sensor is mounted on the part of the quickdraw towards the ground: in this way, when the quickdraw swings, the amount of swinging is higher than if the quickdraw is mounted towards the ceiling. This consistent orientation aids in maintaining the reliability and accuracy of the sensor data, as corroborated by other studies on sensor calibration in sports settings [31].

### 6.3. Participant Variability

As we describe in Section 3, the specific data collection to develop the approach described here involved only one climber. However, we have to add that the key observation allowing this work, i.e., that, when the climber is lowering, the first quickdraw is tilted upward because of the tension that the weight of the climber causes on the rope, was made throughout the duration of the research project of which this paper was part of, with many different climbers of different skill levels, body sizes, and climbing styles. We conclude that the orientation of the quickdraw during lowering is independent from physical properties or the skills of the climber.

Given that lowering is the phase where the ascending activity and interaction of the climber with the holds and routes on the wall are finished, factors such as the climber’s best climb and injury history were not considered in this study because they do not influence the lowering activity. From a safety point of view, the most important aspect to consider is the weight difference between the climber and the belayer [31,32]. This aspect, while critical for safety, was beyond the scope of this paper.

### 6.4. Benefits of Our Multidisciplinary Feature Engineering Approach

Classification approaches based on machine learning algorithms such as logistic regression, random forest, and k-nearest neighbors are simple to develop and can classify multiple activities. ANNs are powerful and highly accurate; they can be slow to train and complex to implement. Real-time execution of these algorithms can be challenging due to the computational demands of processing high-dimensional feature spaces and the trade-off between computational load and precision in defining activity borders [2]. These approaches often require an extra step for feature selection, which can be challenging.

In our approach, we decided on a precise feature—the orientation of the smart quickdraw by integrating mechanical and robotics-related knowledge along with our observations in the climbing gym. This allowed us to skip the feature selection step, making our method more efficient than other machine learning methods. By focusing on a single, well-chosen feature, we could use a simple decision tree model, which is advantageous compared to more complex deep learning and neural network-based approaches.

In this work, by introducing our method, we aim not only to present a good feature for detecting the lowering phase but also to emphasize the importance of feature engineering using multidisciplinary science in machine learning and deep learning. This approach highlights the benefits of combining domain-specific knowledge with machine learning to enhance performance and simplify implementation.

The detected lowering activity described here helps to understand when a climb has ended. In a setup where data are constantly collected, this is useful for dividing data into episodes and reasoning over these episodes, characterizing them with additional metrics such as the number of falls, number of pauses, speed of the ascent, and so on. Typically, after one climb, it is polite to hand over the line to other climbers waiting for their turn. This means that, after one climbing episode, another person is typically climbing. This is an important fact to consider when applying machine learning to data collected about human movements, as it impacts the interpretation and segmentation of climbing activity data.

## 7. Conclusions

In this work, we utilized three-axis accelerometer sensors operating in ultra-low power mode and attached them to the quickdraws hanging from climbing walls. Features were extracted from the sensor at the lowest position while a climber ascended the same line consisting of three routes, 24 times over two different days. Since lowering is not influenced by the skill level of the climber or the difficulty of the route, we believe that this excludes all confounding factors, making the amount of data collected in our study statistically sufficient to analyze the lowering phase, which is a relatively simple activity. However, we acknowledge that having more data is always beneficial in machine learning, and increasing the dataset size can be considered for future work.

Machine learning can enhance the recognition of activities and assessment of performance in sport climbing. While approaches like logistic regression and random forest are simple and effective, ANNs and CNNs, despite their power, can be complex and slow to train. Real-time execution can also be challenging due to the computational demands of processing high-dimensional features. Our methodology focuses on reducing complexity by selecting a key feature—the orientation of the s-qd—and employing a straightforward decision tree model. This approach lowers model complexity and shortens execution time, making it ideal for real-time applications in climbing gyms. Real-time analysis is essential for identifying high-risk situations, thereby enhancing safety and performance. Additionally, our approach emphasizes the benefits of multidisciplinary feature engineering, combining domain-specific knowledge with machine learning to improve performance and simplify implementation.

From each measured sample, orientation was calculated in the 3D plane. The statistical features obtained from the orientation of the s-qd used for classification show that the lowering activity is detected with a precision greater than 90% using orientations of the s-qd. These results align with our visual observations in climbing gyms, where the first s-qd was oriented upward all the time during lowering. This is due to the difference in the tension of the rope from above (climber) and below (belayer). 

## Data Availability

The data that support the findings of this study are not publicly available due to privacy and ethical restrictions. The data contain information that could compromise the privacy of the participants.

## Abbreviations

The following abbreviations are used in this manuscript:

s-qd	Smart-quickdraws
ADC	Analog-to-digital converter
FRSD	French rating scale of difficulty
MCU	Microcontroller unit
API	Application programming interface
2D	Two-dimensional
3D	Three-dimensional
ANN	Artificial Neural Network
CNN	Convolutional Neural Network
